# The SARS‐CoV‐2 Omicron (B.1.1.529) variant and the re‐emergence of COVID‐19 in Europe: An alarm for Bangladesh

**DOI:** 10.1002/hsr2.545

**Published:** 2022-03-13

**Authors:** Md. Rabiul Islam

**Affiliations:** ^1^ Department of Pharmacy University of Asia Pacific Dhaka Bangladesh

The latest variant named SARS‐CoV‐2 Omicron (B.1.1.529) is the most heavily muted strain discovered so far in one of the provinces in South Africa on November 24, 2021. The World Health Organization (WHO) has designated the newly detected variant as Variant of Concern (VOC) on November 26, 2021.^1^ After Alpha, Beta, Gamma, and Delta, it is the fifth VOC of coronavirus. The distinguishing characteristic of Omicron is the prime immunogenic target of antibodies triggered by immunization or through natural infections, which can hold an astonishingly huge number of genetic alterations (more than 50), of which nearly 30 mutations are on the spike (S) membrane protein, most likely to aggregate around the site of receptor‐binding motif; the consensus sequence of this variant has 44 amino acid changes, deletion of 6 amino acids, and insertion of 1 amino acid when compared with the reference strain of SARS‐CoV‐2.^2^ Europe has become the epicenter of the COVID‐19 pandemic again due to the re‐emergence of COVID‐19 cases in European countries. According to WHO, the highly contagious Delta variant has just two mutations to the receptor‐binding domain that accounts for more than 99% of COVID‐19 infections of the World before the introduction of Omicron. Following South Africa, more than 150 countries have been detected with Omicron variant as of January 20, 2022,^3^ and the United Kingdom reported the first known death of a patient with Omicron variant.^4^ The newly emerged variant has arrived in Europe during the devastating fourth wave of COVID‐19 upsurge due to the Delta variant. As of January 28, 2022, Europe logged more than 138 million COVID‐19 cases, which is the highest across the region followed by the Americas (more than 132 million confirmed cases).^5^ Also, Europe is currently representing 38% of all COVID‐19 cases reported globally.^5^ In Europe, people gather together in the run‐up to the religious festival Christmas during the early winter season; this is when the new variant Omicron had arrived, hence this season might have provided a perfect ground for mass transmission of the virus.

The COVID‐19 symptoms due to the Omicron variant are milder so far. However, the Omicron variant could potentially raise death rates due to its very high transmissibility.^6^ People suffer flu‐like symptoms due to the Omicron variant. The major symptoms for the Omicron variant are fever, sore throat, cough, weariness, and aches, whereas low oxygen saturation, abnormal pulse rates, and shortness of breath are frequently reported symptoms for the Delta variant.^7^ Moreover, tiredness, sore throat, muscle or body aches, loss of taste or smell, runny nose were common for earlier variants.^8^ We do not have enough epidemiological data about the Omicron variant yet. However, some early studies suggest that the illness due to the Omicron variant is mild so far.^8^ The findings by research in England, Scotland, and South Africa stated that the omicron variation has a 15%−80% reduced risk of hospitalization than the Delta variant.^9^, ^10^ Approximately, 15%−20% infected individuals with the Omicron variant may require to visit the hospital, which is a 40%−45% lower probability of hospitalization than those infected with the Delta variant.^11^ According to new data from South Africa, during October and November of 2021, Omicron infection shows an 80% lower count in people with other variants having a lesser likelihood of being admitted to the hospital.^12^

According to preliminary data by the Health Security Agency of the United Kingdom, two doses of AstraZeneca's nCoV‐19 or BioNTech's BNT162b2 vaccines are effective against symptomatic infection.^13^ However, the increased rate of infections among the people who were previously infected or have received double vaccine shots has created new worries across the countries.^14^, ^15^, ^16^, ^17^ Pfizer and BioNTech reported that three doses of their vaccine are capable of eliminating the new Omicron variant in certain lab tests, and additional booster doses will provide additional protection.^18^ A recent Pfizer research claimed that vaccination protection after 6 months dropped from 96.2% to 83.7%.^19^ According to their findings, the Pfizer (two doses) vaccine provides more than 80% immunity against severe illness and death, while additional boosters might increase the immunity by 10%. Moreover, US‐FDA authorized three antiviral drugs for emergency use in treating COVID‐19 patients. Remdesivir is a nucleotide that inhibits the RNA‐dependent polymerase enzyme of SARs, which is present in the epithelial cells of the airways in humans; it shows significant nanomolar action.^20^ Moreover, molnupiravir shows synergistic effects with other antivirals according to preclinical studies.^21^, ^22^ Also, paxlovid reduces hospitalization or death rate by 89% among patients with mild‐to‐severe COVID‐19 symptoms.^23^ Therefore, these antiviral drugs might be potentially supportive in fighting the new wave of COVID‐19 pandemic due to the Omicron variant along with vaccines.

The first novel coronavirus strain SARS‐CoV‐2 was introduced in Bangladesh through the returnees from the European country, Italy.^24^ After that, Bangladesh fought relentlessly against the uncontrollable transmission of the virus and different variants of SARS‐CoV‐2 like the UK variant, Delta variant, Delta Plus variant, and South African variant in different timeframes.^25^ Since December 2021, the COVID‐19 pandemic situation in Bangladesh was under control due to the mass vaccination drive, imposing movement restrictions and lockdowns at different intervals, and raising public awareness about coronavirus.^26^ Bangladesh logged zero single‐day death from COVID‐19 on November 20, 2021, for the first time since April 3, 2020.^27^ As of January 24, 2022, Bangladesh has administered more than 156 million vaccine doses among the population.^28^ Assuming that every individual needs two doses of vaccine, it is enough to have vaccinated about 28% of the Bangladeshi. Bangladesh relaxed the last restrictions on public movement and gathering across the country from August 11, 2021. The authority of Bangladesh has reopened all offices, banks, markets, and public transportation. Educational institutions reopened in‐person classes across the country on September 12, 2021, after being shut down for more than one and a half years as fresh COVID‐19 cases had begun to fall.^27^ The country has been witnessing positivity rates below/around 2% for the past few weeks. The overall COVID‐19 fatality rate remained at 1.6%, the overall recovery rate at 97.7%, and the overall positivity lowered to 14.7% in Bangladesh.^28^ The current COVID‐19 situation marks an improvement in the pandemic and hopeful scenario about the containment of the COVID‐19 pandemic in Bangladesh. The mass vaccination rate of this country is still low.^29^ COVID‐19 pandemic and associated burdens are high in Bangladesh as a lower‐middle‐income country.^30^ During the time of Omicron, a lot of migrants had returned home from Europe to Bangladesh for Christmas vacation. Many countries decided to travel ban from southern Africa due to the Omicron variant. However, no decision had been made yet for other countries with confirmed Omicron cases.^31^ The Omicron variant has triggered alarm for countries with poor healthcare infrastructure, crowded populations, and lower vaccination rates.^32^, ^33^ Bangladesh reported the first COVID‐19 patient due to Omicron variant on December 12, 2021.^34^ On January 27, 2022, the country reported more than 15,000 new confirmed COVID‐19 cases.^35^ The present positive test rate is about 30% since January 25, 2022.^35^

Therefore, the government authorities should be more prepared to prevent any further COVID‐19 wave due to this highly mutated and transmissible coronavirus variant.^36^, ^37^, ^38^, ^39^, ^40^ The authority can implement their previous pandemic‐tackling experiences to tackle the new variant successfully. Most importantly, the government should tighten the travel restrictions or regulations of returning passengers. The government of Bangladesh imposed a 14‐day mandatory institutional quarantine upon returning from the African country. However, 240 people have come from South Africa in the last 1 month and the authority is trying “contract tracing” them but they have switched off their mobile phones. Therefore, the healthcare authorities must install the COVID‐19 testing facilities (e.g., RT‐PCR) in the airports to test the passengers on arrival. Prior vaccination and RT‐PCR test report should be made mandatory to enter the country. To curb the transmission of the newly discovered variant, mandatory quarantine of the returnees should be implemented. The mass vaccination program should be further strengthened. Additionally, individuals should take necessary measures to reduce the risk of Omicron variant transmission. The authority must implement proven public health safety guidelines.

## KEYWORDS

B.1.1.529, Bangladesh, COVID‐19, epidemiology, Omicron variant, public health

## CONFLICT OF INTEREST

The author declares no conflict of interest.

## ETHICS STATEMENT

It was an analysis of online available aggregate data. No ethical approval was needed.
